# Prognostic nomogram for adult patients with acute myeloid leukemia: A SEER database analysis

**DOI:** 10.1097/MD.0000000000015804

**Published:** 2019-05-24

**Authors:** Cunte Chen, Peipei Wang, Caixia Wang

**Affiliations:** a Department of Hematology; b Department of Oncology, Guangzhou First People's Hospital, Guangzhou Medical University and Guangzhou First People's Hospital, School of Medicine, South China University of Technology, Guangzhou, Guangdong, China.

**Keywords:** acute myeloid leukemia, cancer-specific survival, nomogram, overall survival, prognosis

## Abstract

Acute myeloid leukemia (AML) is hematopoietic malignancy. This study was designed to develop an individualized prognostic nomogram to predict cancer-specific survival (CSS) and overall survival (OS) of AML.

The clinical data of AML patients (n = 58,882) diagnosed from 1973 to 2014 were obtained from the Surveillance, Epidemiology, and End Results database. The patients were divided into training cohort (n = 29,441) and validation cohort (n = 29,441). The prognostic nomograms were designed with clinical variables selected by multivariate Cox regression model in training cohort. The concordance index (C-index), calibration curve, and receiver operating characteristic curve were used to assess the performance of the nomograms.

The predictors in nomogram for CSS were AML subtypes, age, sex, region, marital status, and chemotherapy, whereas the predictors for OS were AML subtypes, age, sex, region, race, marital status, and chemotherapy. The C-indexes of the nomograms in internal validation for CSS and OS were 0.712 and 0.703, respectively, whereas the C-indexes in external validation for CSS and OS were 0.712 and 0.705, respectively. The area under the curve of receiver operating characteristic curves for CSS and OS were 0.799 (95% confidence interval: 0.792–0.806) and 0.809 (95% confidence interval: 0.803–0.816), respectively.

The individualized prognostic nomogram could perform relatively accurate prediction of outcome in adult patients with AML.

## 1 Introduction

Acute myeloid leukemia (AML) is a highly heterogeneous hematological malignant disease derived from myeloid hematopoietic progenitor cells^[1]^ and the most common type of myeloid malignancy in adults with an incidence of 3.7 per 100,000 persons.^[2]^ The clinical outcome of AML patients are closely related to immune, molecular, and cytogenetic abnormalities,^[3–5]^ as well as age at diagnosis, sex, marital status, insurance status, and county-level income.^[6–8]^ Over the past few decades, diagnosis and treatment in patients with AML has improved, but the overall survival (OS) rate for AML is still low, less than 50%.^[9]^ Therefore, prognostic models need to be established to provide evidence for diagnosis and treatment of AML in clinic.

The nomogram models have been validated in the prognosis of several malignancies, which can provide good statistical predictions on survival probability.^[10–12]^ Recent research shows that nomogram models are built to analyze OS by integrating mutated genes for older patients with AML.^[13]^

In this study, we tried to design a nomogram model for predicting the survival probability of adult patients with AML, using the Surveillance Epidemiology and End Result (SEER) dataset between 1973 and 2014. The SEER program in the National Cancer Institute's Division of Cancer Control and Population Sciences is the most reliable and comprehensive source of population-based cancer information in the United States, which provides a large dataset for our nomogram models construction. AML subtypes, sex, age at diagnosis, region, race–ethnicities, marital status, and chemotherapy in SEER program were included into the nomogram models analysis. The visual format of the nomogram helps to understand the prognosis of an individual so that their physicians can make a corresponding treatment based on the prognosis.

## 2 Materials and methods

### 2.1 Data sources

The SEER program, involving in approximately 26% of the US population, is a publicly available database and primary source of cancer statistics that is supported by the Surveillance Research Program in the National Cancer Institute's Division of Cancer Control and Population Sciences.^[14]^ The clinical data of AML patients diagnosed from 1973 to 2014 were obtained from the SEER database by using the SEER∗Stat program (version 8.3.5).^[14]^ A total of 65,535 records were obtained. In the SEER data, the AML subtypes were classified according to the 3rd edition of the International Classification of Disease Oncology (ICD-O-3) and WHO 2008 definitions.^[15,16]^ The AML subtypes included in this study are as follows: 9840/3 – acute erythroid leukemia; 9861/3 – AML, NOS; 9865/3 – AML with t (6;9)(p23;q34), DEK-NUP214; 9866/3 –acute promyelocytic leukemia (AML with t (15;17)(q22;q12)) PML/RARA; 9867/3 – acute myelomonocytic leukemia; 9869/3 – AML. inv (3)(q21;q26.2) or t (3;3)(q21;q26.2), RPN1-EVI1; 9871/3 – AML with inv (16)(p13.1q22) or t (16;16)(p13.1;q22), CBFB-MYH11; 9872/3 – AML with minimal differentiation; 9873/3 – AML without maturation; 9874/3 – AML with maturation; 9891/3 – acute monoblastic and monocytic leukemia; 9895/3 – AML with myelodysplasia-related changes; 9896/3 – AML, t (8;21)(q22;q22) RUNX1-RUNX1T1; 9897/3 – AML with t (9;11)(p22;q23), MLLT3-MLL; 9910/3 – acute megakaryoblastic leukemia; 9911/3 – AML (megakaryoblastic) with t (1;22)(p13;q13), RBM15-MKL1; and 9920/3 – therapy-related myeloid neoplasm. Among the above AML subtypes, ICD-O3 codes 9840/3, 9861/3, 9865/3, 9867/3, 9869/3, 9871/3, 9872/3, 9873/3, 9874/3, 9891/3, 9895/3, 9896/3, 9897/3, 9910/3, 9911/3, and 9920/3 belonged to non-APL AML, whereas 9866/3 belonged to APL. The criteria of region were as follows: East includes Connecticut, Atlanta (Metropolitan), and Rural Georgia; Northern Plains include Detroit (Metropolitan) and Iowa; Pacific Coast includes San Francisco Oakland, Hawaii, Seattle (Puget Sound), San Jose-Monterey, and Los Angeles; and Southwest includes New Mexico and Utah.

The following cases were excluded: age at diagnosis <18 years; unknown survival time; unknown marital status; and unknown race/ethnicity. Owing to the small number of patients from Alaska, they might cause bias in survival analysis, so they were also excluded.

The following variables were analyzed: AML subtype, sex, age at diagnosis, region, race/ethnicity, marital status, chemotherapy, cause-specific death, and vital status. It is worth noting that the race/ethnicity of yellow included Chinese, Korean, and Japanese in this study. Additionally, in marital status, married included separated, whereas single included never married or unmarried. According to the prognosis of patients,^[17,18]^ AML was divided into APL and non-APL. The follow-up time was recorded as the duration of time from the diagnosis to death or the last day of survival information documented in the SEER registry. The variable of “vital status recode” was used to determine the status of survive.

**Figure 1:**
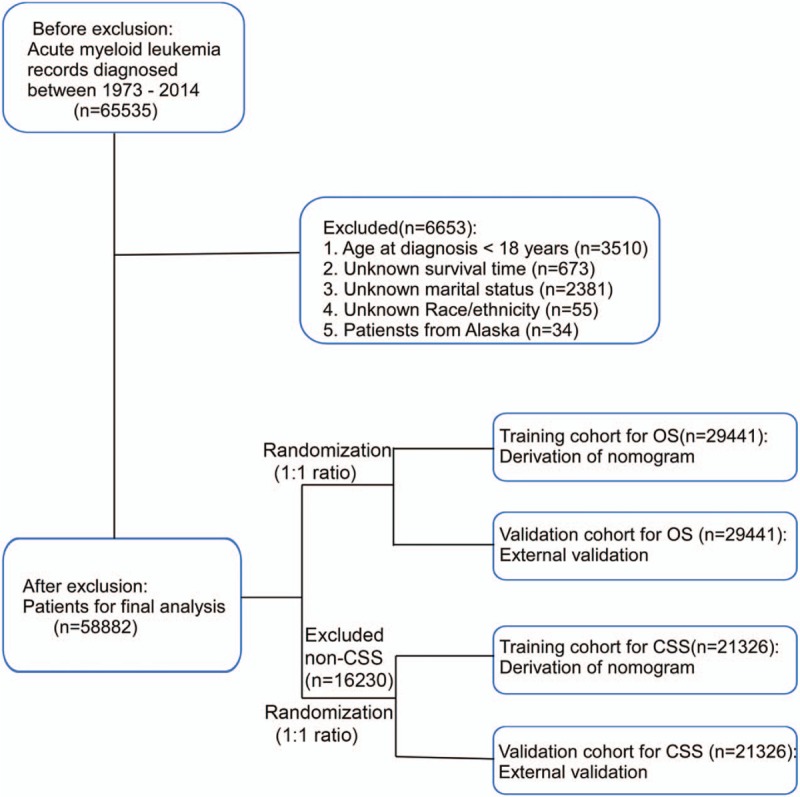
Flow diagram for data selection and research strategy.

After exclusion of patients based on the above criteria, 58,882 AML patients were identified for OS analysis. Furthermore, after excluding patients with noncancer-specific death [noncancer-specific survival (CSS)], 42,652 patients were identified as entering CSS analysis. Ultimately, patients were randomly assigned to a training cohort and a validation cohort (1:1 ratio) for OS and CSS analysis (Fig. 1). The clinical information of adult AML are publicly available in the SEER program, so the approval of local ethics committee was not needed.

### 2.2 Statistical analysis

Qualitative variables were categorized prior to modeling based on clinical experience and significance. For continuous variables, the optimal cutoff of age was obtained using X-tile software version 3.6.1 (Yale University, New Haven, CT).^[19]^ Univariate and multivariate analyses were performed by using the Cox proportional hazard regression models in SPSS Statistical Package version 22.0 (IBM, Chicago, IL) to clarify the independent prognostic value of clinical variables for OS and CSS. Clinically significant variables for OS and CSS, which were selected in multivariate Cox proportional hazard regression models in a backward stepwise manner based on the Akaike information criterion, were assessed for incorporating into the nomogram model. The foreign, rms, hmisc, lattice, survival, formula, and ggplot2 packages in R, version 3.5.1 (http://www.r-project.org/) were applied for nomogram model analysis. Model performance was assessed by internal and external validation, which was performed by discrimination with concordance index (C-index) and calibration curves using 1000 sample bootstrap. Then, all cohorts of patients were given a total score using standard points obtained from the nomogram models, which could predict survival rates of AML patients. The patients were randomly assigned using the Microsoft Excel 2007. The receiver operating characteristic (ROC) curves was used for predictive ability of nomogram in SPSS Statistical Package version 22.0 (IBM, Chicago, IL). A 2-tailed *P* value <.05 was considered to indicate statistical significance. This study was performed in accordance with the ethical principals of the Declaration of Helsinki for medical research involving human participants.^[20]^

## 3 Results

### 3.1 Cohort characteristics

The clinical characteristics of the patients in the training and validation cohorts for OS and CSS analysis were listed in Table 1.

**Table 1:**
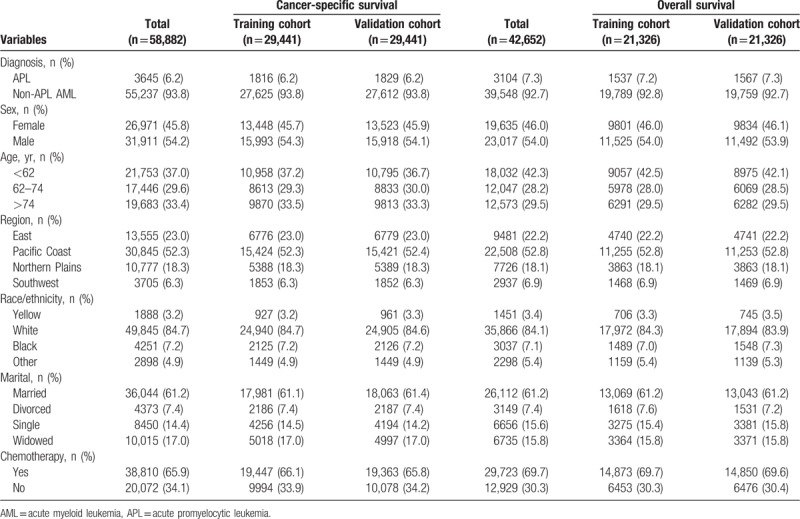
Clinical characteristics of patients with AML.

### 3.2 X-tile for the optimal cutoff of age

**Figure 2:**
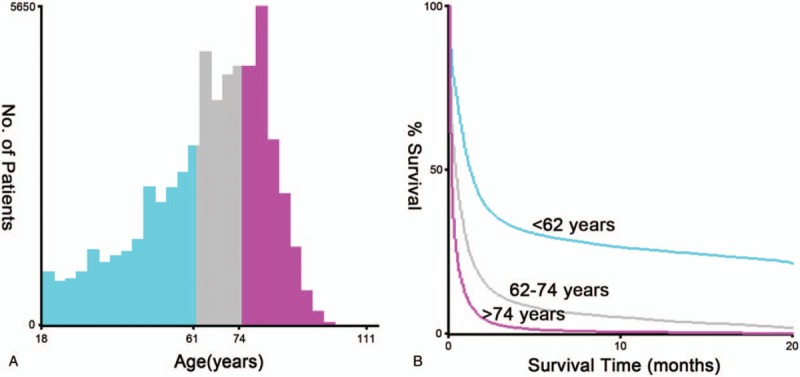
Optimal cutoff value of age obtained from X-tile software. (A) Distribution of the number of patients was showed in different age groups based on the cutoff values. (B) The survival curve was plotted on the basis of the optimal cutoff values, which revealed significant difference.

X-tile software was used to determine the optimal cutoff value of age in total AML patients (n = 58,882) after screening, which was applied for univariate and multivariate Cox proportional hazard regression analysis, as well as nomogram model construction. As shown in Figure 2, the optimal cutoff of age for analysis were <62, 62–74, and >74 years, which indicated significant difference among cutoff values.

### 3.3 Cox regression analysis of training cohort

Univariate Cox proportional hazard regression analysis for OS and CSS suggested that there were significant differences in survival rates of AML subtypes, age, gender, region, race/ethnicity, marital, and chemotherapy, which could be further included in multivariate Cox regression analysis (Table 2). As shown in Table 3, multivariate Cox proportional hazard regression models demonstrated that AML subtypes, age, sex, region, race/ethnicity, marital status, and chemotherapy were independent prognostic factors of AML in the OS analysis, whereas AML subtypes, age, sex, region, marital status, and chemotherapy, except race/ethnicity, were independent prognostic factors of AML in the CSS analysis.

**Table 2:**
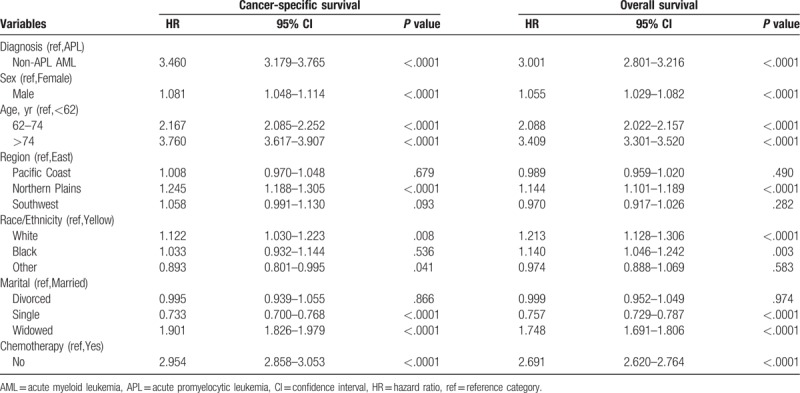
Univariate Cox regression analysis of training cohort.

**Table 3:**
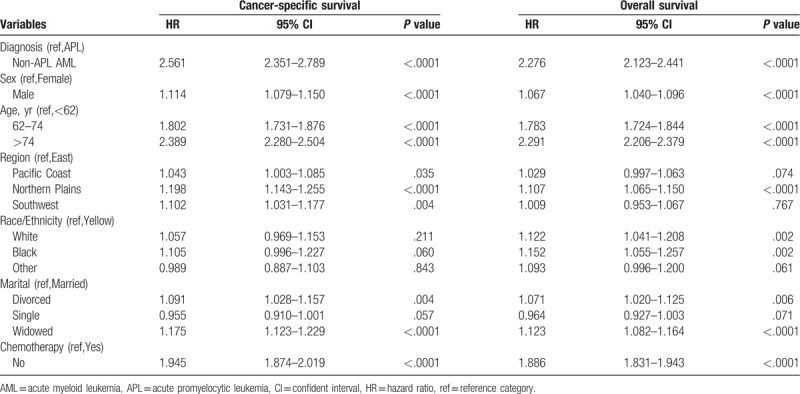
Multivariate Cox regression analysis of training cohort.

### 3.4 Nomograms of AML for CSS and OS

**Figure 3:**
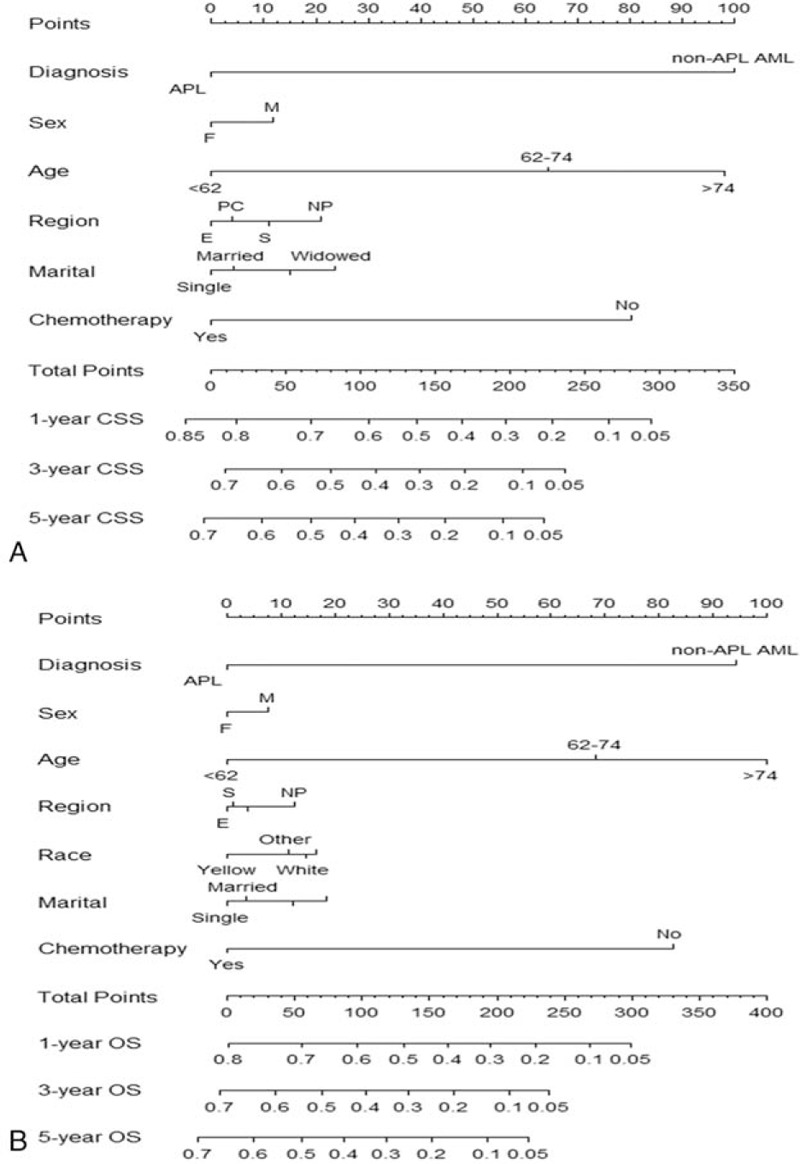
Nomogram models for CSS (A) and OS (B) of AML. First of all, the covariate of each patient was given a point based on the nomogram. Then, the total points were obtained by gathering the given points of all covariates of a patient. Finally, the survival probabilities of 1, 3, and 5-year CSS or OS corresponding to the total points could be showed by the nomogram. Additionally, a higher total point usually suggested a higher possibility of a lower predicted survival probability (CSS or OS). AML = acute myeloid leukemia, CSS = cancer-specific survival, OS = overall survival.

Clinical parameters after multivariate Cox regression selection were channeled into the construction of training cohort nomogram (Fig. 3). However, due to *P* < .05 of multivariate Cox regression in the CSS, race/ethnicity could not be employed in nomogram. Details of the labels for tick marks and points in nomograms were shown in Table 4.

**Table 4:**
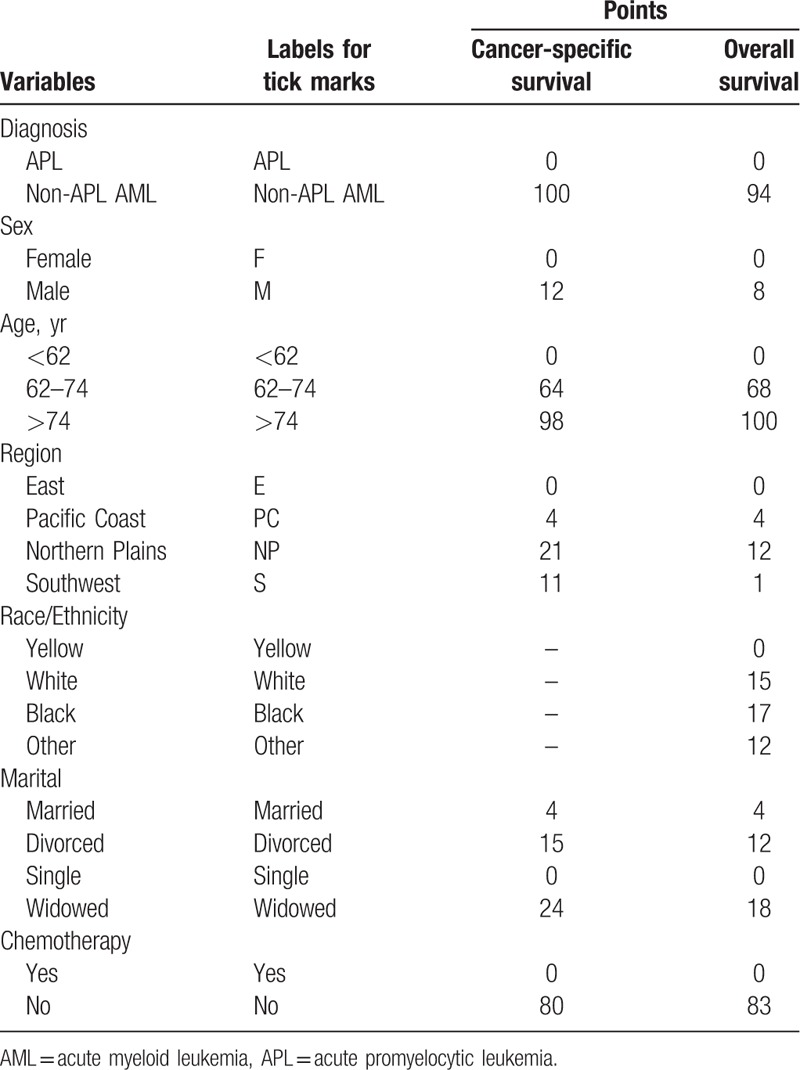
Points for variables in nomograms.

### 3.5 Internal validation

**Figure 4:**
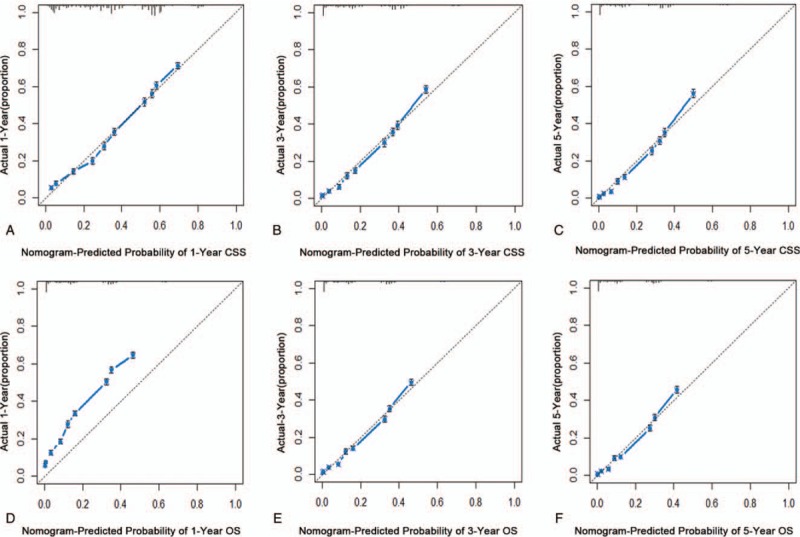
Internal validation of nomograms in the training cohorts. The predicted probabilities of 1, 3, and 5-year CSS (A–C) and OS (D–F) were consistent with the actual survival proportions of patients with AML. AML = acute myeloid leukemia, CSS = cancer-specific survival, OS = overall survival.

The C-indexes of 1000 sample bootstrap were 0.712 and 0.703 for the CSS and OS predictive nomograms, respectively, which indicated that nomograms for CSS and OS showed relatively precise ability of discrimination. Further calibration curves manifested that the probability of predicted 1, 3, and 5-year CSS and OS in nomograms were well consistent between the predicted outcome and actual observation (Fig. 4).

### 3.6 External validation

**Figure 5:**
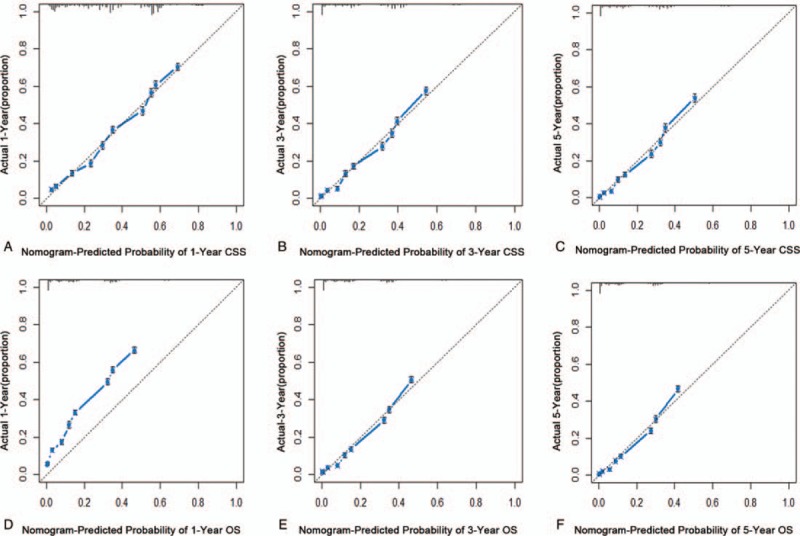
External calibration of nomograms in the validation cohorts. One thousand sample bootstrap calibration was used for external validation cohorts, indicating that the predicted probabilities of 1, 3, and 5-year CSS (A–C) and OS (D–F) were well related with the actual survival proportions. CSS = cancer-specific survival, OS = overall survival.

In the external validation cohort, the C-indexes of predictive accuracy for CSS and OS were 0.712 and 0.705, respectively (Fig. 5). The external calibration curves also illustrated good validation between predicted and observed 1, 3, and 5-year CSS and OS. The discrimination and calibration validation of external cohort definitely certificated that nomogram models in this study could be comparatively accurate enough to predict the CSS and OS rate of patients with AML.

### 3.7 ROC curves for CSS and OS

**Figure 6:**
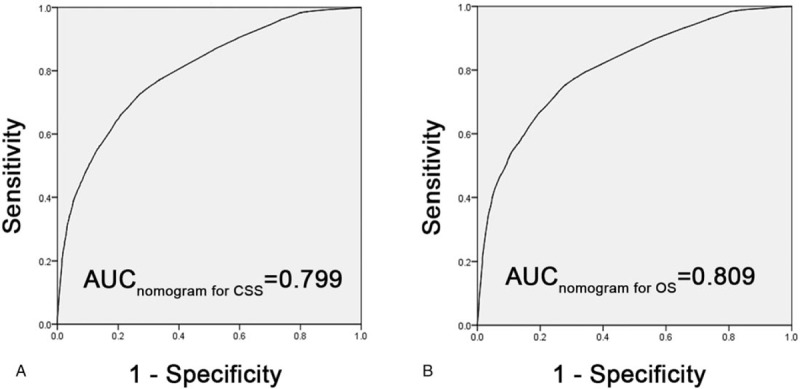
Predictive ability for cancer-specific survival (CSS) and overall survival (OS) in training cohorts. The AUC of ROC curves for CSS and OS were 0.799 (A) and 0.809 (B), respectively. AUC = area under the curve, ROC = receiver operating characteristic.

The predictive ability for CSS and OS in training cohorts is by using ROC curves. The area under the curve (AUC) of ROC curves for CSS and OS were 0.799 [95% confidence interval: 0.792–0.806, Fig. 6(A)] and 0.809 [95% confidence interval: 0.803–0.816, Fig. 6(B)], respectively.

## 4 Discussion

The nomogram model, compared with other predictive models, integrated different clinical variables to offer a more accurate and personalized prognosis assessment system.^[13,21–23]^ In this work, we developed 2 nomogram models based on the SEER database to predict CSS and OS for adult patients with AML. Although the predicted and observed probabilities of 1-year OS in the nomograms were not completely consistent, the C-indexes were all higher than 0.7, which achieved considerable prediction accuracy and repeatability when nomograms were applied to training and validation cohorts. Simultaneously, 3 and 5-year CSS and OS in nomograms showed good predictive accuracy. For the nomogram of AUC, the AUC were consistent with the C-index, indicating that the models could provide a good prognostic assessment system in patients with AML.

Here, some variables in the nomogram models were analyzed. Acute promyelocytic leukemia (APL) was generally characterized by the t (15; 17)(q22; q21) chromosomal translocation to generate *PML-RAR* fusion gene, which was the target site for all-trans retinoic acid. Over the past years, due to the application of all-trans retinoic acid and arsenic trioxide (As_2_O_3_), the clinical complete remission (CR) rate and status of the disease-free survival of APL have been significantly improved, and the CR rate has been higher than 90%.^[17]^ However, 3-year OS rate of non-APL AML was still poor, less than 30%.^[18]^ In the present study, the points of non-APL AML in nomograms for CSS and OS were 100 and 94, respectively, indicating that subtype of AML was a strong predictor of prognosis in nomogram models established by the AML data of the SEER program.

With the prolonged life expectancy, the incidence of AML was rising in the aging population. Over the past few decades, with the great progress making in the diagnosis and treatment of AML, the outcome of young patients has been greatly improved, but the prognosis of elderly patients (>60 years old), whose long-term OS rate is less than 10%, was still very poor.^[18,24]^ The risk ratios of age were more than 1.7 in multivariate Cox regression and the points of age in nomograms for CSS and OS were all more than 60, suggesting that age, especially >74 years old, was a strong predictor of outcome in patients with AML.

AML is hematopoietic malignancy progressing rapidly, whose natural process is of only a few months.^[2]^ However, 50% to 60% patients with AML could achieve CR after intensive induction chemotherapy, and the long-term OS rate after chemotherapy could be improved to 15% to 30%.^[25,26]^ We found that patients without chemotherapy had risk ratios of more than 1.8 and the points in the nomogram were all more than 80, which played an important role in predicting the outcomes of patients.

Studies have shown that sex, region, and marital status were predictors of outcomes in AML patients,^[7,27]^ which were consistent with our findings. However, compared with AML subtype, age, and chemotherapy, the points of sex, region, and marital status in nomogram were low, showing that the predictive ability was relatively poor.

However, it is worth noting that population-based data of SEER program usually does not include detailed clinical data such as white blood cell,^[28]^ relapse,^[29]^ and risk stratification,^[18]^ which may help to improve the reliability and accuracy of the nomogram models. Hence, larger clinical data was needed to validate the accuracy and repeatability of the nomogram models in the future.

Overall, in this study, the bootstrap-corrected and ROC curve-validated nomogram models could perform comparatively accurate prediction of 1, 3, and 5-year survival probabilities, which were clinically practical and relatively reliable in adult patients with AML. However, an independent external validation data will still be required to validate the nomogram models in the future, making the models more reliable.

## Author contributions

**Conceptualization:** Caixia Wang.

**Data curation:** Cunte Chen, Peipei Wang.

**Formal analysis:** Cunte Chen, Peipei Wang.

**Funding acquisition:** Caixia Wang.

**Methodology:** Cunte Chen, Peipei Wang.

**Supervision:** Caixia Wang.

**Writing – original draft:** Cunte Chen, Peipei Wang.

**Writing – review & editing:** Cunte Chen, Peipei Wang, Caixia Wang.

## References

[1] Elihu E Hartmut DH Acute myeloid leukaemia. Lancet 2006;71:1894–907. 10.1016/S0140-6736(06)69780-8 17126723

[2] Deschler B Lubbert M Acute myeloid leukemia: epidemiology and etiology. Cancer 2006;107:2099–107. 17019734 10.1002/cncr.22233

[3] Ostronoff F Othus M Lazenby M Prognostic significance of NPM1 mutations in the absence of FLT3-internal tandem duplication in older patients with acute myeloid leukemia: a SWOG and UK National Cancer Research Institute/Medical Research Council report. J Clin Oncol 2015;33:1157–64. 25713434 10.1200/JCO.2014.58.0571 PMC4372852

[4] Leong SR Sukumaran S Hristopoulos M An anti-CD3/anti-CLL-1 bispecific antibody for the treatment of acute myeloid leukemia. Blood 2016;129:609–18. 27908880 10.1182/blood-2016-08-735365 PMC5290988

[5] Bochtler T Granzow M Stölzel F Marker chromosomes can arise from chromothripsis and predict adverse prognosis in acute myeloid leukemia. Blood 2017;129:1333–42. 28119329 10.1182/blood-2016-09-738161

[6] Hossain MJ Xie L Sex disparity in childhood and young adult acute myeloid leukemia (AML) survival: evidence from US population data. Cancer Epidemiol 2015;39:892–900. 26520618 10.1016/j.canep.2015.10.020 PMC4679651

[7] Borate UM Mineishi S Costa LJ Nonbiological factors affecting survival in younger patients with acute myeloid leukemia. Cancer 2015;121:3877–84. 26367383 10.1002/cncr.29436

[8] Appelbaum FR Gundacker H Head DR Age and acute myeloid leukemia. Blood 2006;107:3481–5. 16455952 10.1182/blood-2005-09-3724 PMC1895766

[9] Ferrara F Unanswered questions in acute myeloid leukaemia. Lancet Oncol 2004;5:443–50. 15231251 10.1016/S1470-2045(04)01512-8

[10] Rose PG Java J Whitney CW Nomograms predicting progression-free survival, overall survival, and pelvic recurrence in locally advanced cervical cancer developed from an analysis of identifiable prognostic factors in patients from NRG Oncology/Gynecologic Oncology Group randomized trials of chemoradiotherapy. J Clin Oncol 2015;33:2136–42. 25732170 10.1200/JCO.2014.57.7122 PMC4477785

[11] Liang W Zhang L Jiang G Development and validation of a nomogram for predicting survival in patients with resected non–small-cell lung cancer. J Clin Oncol 2015;33:861–9. 25624438 10.1200/JCO.2014.56.6661

[12] Han DS Suh YS Kong SH Nomogram predicting long-term survival after d2 gastrectomy for gastric cancer. J Clin Oncol 2012;30:3834–40. 23008291 10.1200/JCO.2012.41.8343

[13] Wang J Ma Z Wang Q Prognostic utility of six mutated genes for older patients with acute myeloid leukemia. Int J Cancer 2018;142:1664–70. 29193057 10.1002/ijc.31178

[14] SEER. SEER∗Stat 8.3.5 Database. 2018 Available at: https://seer.cancer.gov/ Accessed July 4, 2018.

[15] Seer NCI. The Seer Program Coding and Staging Manual 2007. Revised September 2008.

[16] He GS Wu DP Sun AN The 2008 revision of the World Health Organization (WHO) classification of MDS. Chin J Prac Internal Med 2010.

[17] Shen ZX Shi ZZ Fang J All-trans retinoic acid/As_2_O_3_ combination yields a high quality remission and survival in newly diagnosed acute promyelocytic leukemia. Proc Natl Acad Sci U S A 2004;101:5328–35. 15044693 10.1073/pnas.0400053101 PMC397380

[18] Doria-Rose VP Harlan LC Stevens J Treatment of de novo acute myeloid leukemia in the United States: a report from the Patterns of Care program. Leuk Lymphoma 2014;55:2549–55. 24467221 10.3109/10428194.2014.885517

[19] Camp RL Dolledfilhart M Rimm DL X-tile: a new bio-informatics tool for biomarker assessment and outcome-based cut-point optimization. Clin Cancer Res 2004;10:7252–9. 15534099 10.1158/1078-0432.CCR-04-0713

[20] Mihajlovic-Madzarevic V Appendix C: World Medical Association Declaration of Helsinki Ethical Principles for Medical Research Involving Human Subjects. Hoboken, NJ: John Wiley & Sons Inc; 2010.

[21] Yang Y Zhang YJ Zhu Y Prognostic nomogram for overall survival in previously untreated patients with extranodal NK/T-cell lymphoma, nasal-type: a multicenter study. Leukemia 2015;29:1571–7. 25697894 10.1038/leu.2015.44

[22] Wierda WG O’Brien S Wang X Prognostic nomogram and index for overall survival in previously untreated patients with chronic lymphocytic leukemia. Blood 2007;109:4679–85. 17299097 10.1182/blood-2005-12-051458

[23] Wang Y Li J Xia Y Prognostic nomogram for intrahepatic cholangiocarcinoma after partial hepatectomy. J Clin Oncol 2013;31:1188–95. 23358969 10.1200/JCO.2012.41.5984

[24] Farag SS Archer KJ Cancer and Leukemia Group B 8461. Pretreatment cytogenetics add to other prognostic factors predicting complete remission and long-term outcome in patients 60 years of age or older with acute myeloid leukemia: results from Cancer and Leukemia Group B 8461. Blood 2006;108:63–73. 16522815 10.1182/blood-2005-11-4354 PMC1895823

[25] Löwenberg B Ossenkoppele GJ van Putten W High-dose daunorubicin in older patients with acute myeloid leukemia. N Engl J Med 2009;361:1235–48. 19776405 10.1056/NEJMoa0901409

[26] Burnett AK Milligan D Goldstone A The impact of dose escalation and resistance modulation in older patients with acute myeloid leukaemia and high risk myelodysplastic syndrome: the results of the LRF AML14 trial. Br J Haematol 2009;145:318–32. 19291085 10.1111/j.1365-2141.2009.07604.x

[27] Acharya UH Halpern AB Wu QV Impact of region of diagnosis, ethnicity, age, and gender on survival in acute myeloid leukemia (AML). J Drug Assess 2018;7:51–3. 30034924 10.1080/21556660.2018.1492925 PMC6052412

[28] Cassileth PA Sylvester LS Bennett JM High peripheral blast count in adult acute myelogenous leukemia is a primary risk factor for CNS leukemia. J Clin Oncol 1988;6:495–8. 3162514 10.1200/JCO.1988.6.3.495

[29] Rowe JM Tallman MS How I treat acute myeloid leukemia. Blood 2010;116:3147–56. 20558611 10.1182/blood-2010-05-260117

